# Relationships between above- and below-ground carbon stocks in mangrove forests facilitate better estimation of total mangrove blue carbon

**DOI:** 10.1186/s13021-021-00172-9

**Published:** 2021-03-17

**Authors:** Yuchen Meng, Jiankun Bai, Ruikun Gou, Xiaowei Cui, Jianxiang Feng, Zheng Dai, Xiaoping Diao, Xiaoshan Zhu, Guanghui Lin

**Affiliations:** 1 Key Laboratory for Earth System Modeling, Ministry of Education, Department of Earth System Science, Tsinghua University, Beijing, 100084, China; 2 Institute of Ocean Engineering, Tsinghua Shenzhen International Graduate School, Shenzhen, 518055 Guangdong China; 3 School of Marine Sciences, Sun Yat-Sen University, Zhuhai, 510275 Guangdong China; 4 Kunming Institute of Survey and Design, State Forestry and Grassland Administration, Kunming, 650216 Yunnan China; 5 College of Life Science, Hainan Normal University, Haikou, 571158 Hainan China; 6 State Key Laboratory of South China Sea Marine Resource Utilization, Hainan University, Haikou, 570228 Hainan China

**Keywords:** Carbon stock estimation, Mangrove biomass, Coastal wetland ecosystem, Blue carbon, Remote sensing

## Abstract

**Background:**

Although great efforts have been made to quantify mangrove carbon stocks, accurate estimations of below-ground carbon stocks remain unreliable. In this study, we examined the distribution patterns of mangrove carbon stocks in China and other countries using our own field survey data and datasets from published literature. Based on these data, we investigated the possible relationships between above-ground carbon stock (AGC) and below-ground carbon stock (BGC) for mangrove forests, aiming to provide a scientific basis for estimation of total mangrove carbon stocks.

**Results:**

The average above-ground carbon stock in each region was sizeable (ranging from 12.0 to 150.2 Mg/ha), but average below-ground carbon stock was dominant (ranging from 46.6 to 388.6 Mg/ha), accounting for 69–91% of total carbon stock at the sites studied in China. Significant positive relationships were found between above-ground and below-ground mangrove carbon stocks, with the best fitting equation as BGC = 1.58 * AGC + 81.06 (Mg/ha, R^2^ = 0.62, p < 0.01, n = 122) for China. Such linear relationships vary for mangrove forests of different types and locations, from different geographical regions in China to other countries worldwide.

**Conclusion:**

The positive relationship we found between above- and below-ground carbon stocks of mangrove forests in China and worldwide can facilitate more accurate assessments of mangrove blue carbon stocks at regional or global scales using modern techniques including remote sensing.

**Supplementary Information:**

The online version contains supplementary material available at 10.1186/s13021-021-00172-9.

## Background

Mangrove wetlands are highly biodiverse and productive ecosystems, which provide many ecological system services. One of them is the high carbon sink (also called “blue carbon”) capacity for atmospheric CO_2_ in mangrove forests worldwide, resulting from particular seasonal patterns of net ecosystem exchange and unusually high carbon stocks, or gross ecosystem production (GEP) rates, compared with nearby tropical and subtropical forests and wetlands [2, 12, 13, 15, 30, 33, 54, 63, 100]. More than 10% of terrestrial particulate carbon, including dissolved organic carbon (DOC), is exported into the ocean through mangroves [32], even though mangrove forests only occupy 0.5% of global coastal area [3]. Mangroves are highly threatened and more than 35% of the mangrove area has been lost since the 1980s [31]. Despite the small footprint forward of restoration, mangroves still contributes 10% of carbon emissions from deforestation (due to their rapid destruction and high carbon values). Accurate assessments of blue carbon, especially the carbon pool of mangrove ecosystems, will support efforts to control greenhouse gas emissions and mitigate global climate change [33, 75].

An ecosystem’s carbon stock mainly includes vegetation biomass and soil carbon stock [43]. In mangroves, carbon is stored primarily in sediments rather than tree biomass [2, 22, 33, 50, 68]. Mangrove carbon stock is closely related to local biogeochemical and ecological processes [47, 78]. Species diversity, tree density, forest age, and disturbance levels all greatly affect the distribution pattern of mangrove carbon stock components [6, 76]. Communities of mangrove forests shows high spatial heterogeneity, which further hinders the estimation of mangrove carbon stock and confounds the relationship between carbon stock components across spatial scales.

Previous assessments of mangrove carbon stock were mainly limited to a specific location or a certain carbon stock component (biomass carbon or soil carbon). Benefiting from the convenience of large-scale monitoring using the remote sensing technology, more attentions have been paid to monitor the mangrove vegetation, representing the above-ground biomass carbon stock [45, 46, 79, 104]. However, estimation of below-ground soil (or ecosystem) carbon stock remains insufficient. Traditional plot surveys can accurately reveal the distribution of ecosystem carbon stocks. Due to the harsh field conditions of mangrove forests, investigation using the traditional plot method is time-consuming. Thus, assessments of below-ground mangrove soil and ecosystem carbon stocks are generally limited to small-scale regions [71, 76, 81, 101]. Most studies relied on the data from literature or combined models to assess the ecosystem carbon stock [9, 33, 39, 59, 70, 87]. However, there might be great variations and unreliability between different assessments. Components of mangrove carbon stocks can only be roughly estimated using empirical average values and conversion coefficients. In addition, accurate estimation of total mangrove carbon stock is limited due to the unclear relationship between above- and below-ground carbon stocks [34, 73].

Above- and below-ground carbon stocks are closely related in the ecosystem carbon cycle. Plants absorb carbon dioxide from the atmosphere during photosynthesis and store carbon in their stems, branches, and roots. Then, proportional carbon transfers soils [8]. Up to date, most research on mangrove carbon stock mainly focused on the distribution of carbon stock or single carbon stock component, such as the distribution of mangrove carbon stock in a specific range [20, 26, 82, 102], the distribution of mangrove soil carbon stock [10, 20, 90], estimation of above-ground biomass [11, 69], and the distribution of above- and below-ground vegetation biomass [21, 58]. According to the characteristic analysis of stable isotopes in mangrove forests, nearly 60% of the carbon stock in mangrove soil is related to vegetation productivity (such as root biomass) [4]. Furthermore, in response to tidal flooding and increased salinity, mangroves increased carbon allocation to the below-ground portion [83]. However, only few studies have tried to investigate the relationships between above- and below-ground carbon stock for mangrove forests [33, 81]. For example, Donato showed a weak ecosystem carbon correlation in Indonesian mangroves, but further analysis and discussion were missing. Hence, research considering the soil and below-ground biomass carbon stock as a whole is still lacking, and the relationship between the whole below-ground carbon stock and the above-ground biomass carbon, as well as the potential influencing factors require further exploration.

The objectives of this study includes (1) to explore the possible relationships between mangrove above- and below-ground carbon stocks with different vegetation characteristics, geographical conditions, and spatial scales; and (2) to investigate the potential impacts of biological and geographical factors on the relationships between above- and below-ground carbon stocks. We first hypothesize that mangrove above- and below-ground carbon stocks tend to increase consistently with diversity among different communities. To test this hypothesis, we surveyed and analyzed mangrove carbon stock components based on the data from over 251 mangrove forest plots across representative mangroves in China and from the global literature. Different regression models were performed to determine the relationship between carbon stock components and their changes with environmental factors. Our study provides a new approach for more accurate estimation of mangrove carbon stock based on the more precise models between above- and below-ground carbon stocks. In addition, our results will facilitate the mangrove conservation and blue carbon management.

## Materials and methods

### Study sites

**Fig. 1:**
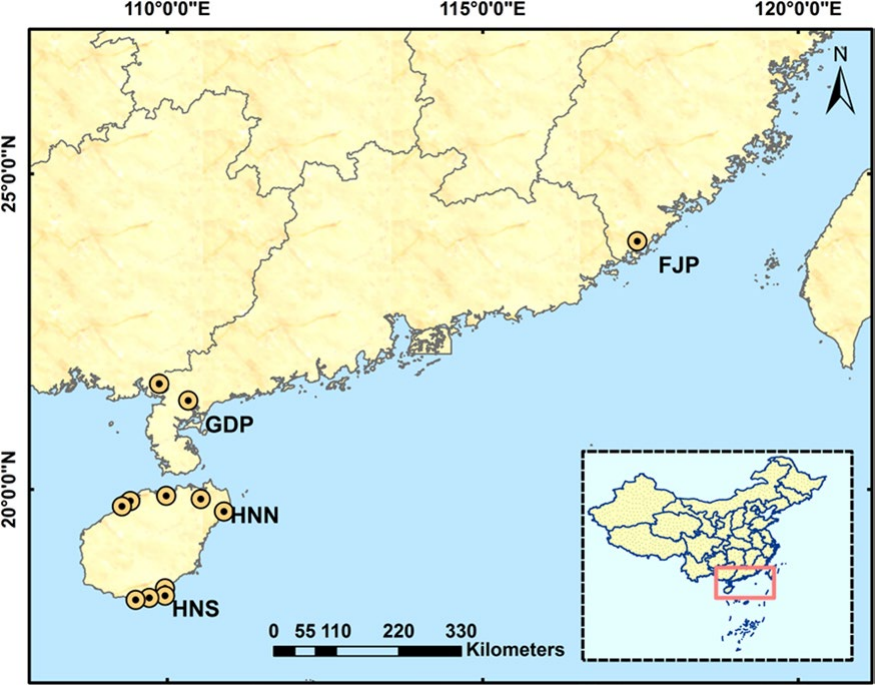
Locations and sampling sites of mangrove communities in mangrove natural reserves at different latitudes in China (*FJP* Fujian Province, *GDP* Guangdong Province, *HNN* Hainan North Region, *HNS* Hainan South Region)

Mangrove ecosystems are mostly distributed along the coastline of southeast China. Based on this feature, we set the field survey sites in four latitude regions: Hainan southwest region (HNS, 18° N–19° N), Hainan northeast region (HNN, 19° N–20° N), Guangdong province (GDP, 20° N–22° N), and Fujian province (FJP, 23° N–24° N) (Fig. 1). According to the climate regionalization scheme in China [105], FJP belongs to the humid south-subtropical zone, both GDP and HNN are situated in the humid marginal-tropic region, and HNS is located in the humid mid-tropic region. Within these four latitude regions, we screened twelve representative mangrove reserves and selected different representative core areas for sampling, based on the characteristics of mangrove distribution in each protected area. Details about the study reserves, area, established time, positions, mangrove species, and descriptions can be found in Table 1.

**Table 1:** Key information for the study sites in China with direct measurements (additional information in Additional file 1: Table S1)

Province	Site name*	Reserve abbreviation	Core mangroves area (ha)	Established time	Latitude	Longitude	Number of field survey plots	Number of dominant species
Fujian	Jiulongjiang, NNR	FZJ	167	2003	23° 53′ N–23° 56′ N	117° 24′ E–117° 30′ E	15	6
Guangdong	Zhanjiang (Gaoqiao), NNR	ZJG	7228	1997	21° 09′ N–21° 34′ N	109°44′E-109°56′E	9	25
	Zhanjiang (Leizhou), NNR	ZJL			20° 48′ N–21° 07′ N	110° 06′ E–110° 30′ E	8	25
Hianan (Northeast)	Chengmai, CNR	HCW	150	1995	19° 54′ N–19° 54′ N	109° 59′ E–109° 59′ E	3	12
	Dongzhaigang, NNR	DZG	1771	1980	19° 51′ N–20° 01′ N	110° 32′ E–110° 37′ E	36	32
	Wenchang, PNR	QLG	1223.3	1988	19° 15′ N–20° 09′ N	110° 30′ E–110° 02′ E	27	33
Hianan (Southwest)	Lingao/Danzhou, NNR	HXY	126.9	1992	19° 49′ N–19° 51′ N	109° 12′ E–109° 34′ E	9	18
	Danzhou, CNR	XYG	79.1	2008	19° 44′ N	109° 17′ E	3	14
	Lingshui, NNR	HLS	120.51	2018	18° 25′ N	109° 58′ E	3	18
	Sanya, CNR	SYH	20	1992	18° 19′ N–18° 37′ N	108° 36′ E–109° 46′ E	3	13
		TLG	4		18° 15′ N–18° 17′ N	109° 42′ E–109° 44′ E	3	11
		QMG	63.3		18° 15′ N	109° 30′ E	3	16

Sources: China Mangrove Conservation Network (CMCN) and the Reserve Official Website

Due to the large discontinuous distribution of mangroves in Guangdong zhanjiang mangrove national nature reserve ZJ(G/L), Two representative separated mangroves were selected in Gaoqiao (109° 44′ E–109° 56′ E, 21° 9′ N–21° 34′ N,ZJG) and Leizhou (110° 6′ E–110° 30′ E, 20° 48′ N–21° 7′ N, ZJL), the core areas of the reserve for research. Hainan Province Southeast (HNS, 18° N–19° N), Hainan Province Northwest (HNN, 19° N–20° N), Guangdong Province (GDP, 20° N–22° N), Fujian Province (FJP, 23° N–24° N)

^*^NNR National Mangrove Nature Reserve, *PNR* Provincial Mangrove Nature Reserve, *CNR* City Mangrove Nature Reserve

### Field sampling

We selected 12 representative protected areas within the latitudes of four mangrove reserves in China. In each protected area, we set several sample plots based on the distribution of mangroves, either continuous or intermittent. Most plots measured 10 m × 10 m area, but a few were slightly adjusted according to mangrove density. In total, 122 mangrove plots were investigated (Table 1) and over 251 plots were established for observation and confirmation of representativeness. Field sampling and assessments were conducted during 2015–2019. We used published mangrove allometric growth equations and wood density specific to the local areas to calculate the above- and below-ground tree biomass for each individual tested. The selection, design of sampling area, and related acquisition measurements were adjusted based on globally applicable mangrove ecosystem carbon stock assessment protocols [49].

In each plot, we recorded all tree vegetation indicators, including species, counts, canopy density, live/dead status, and height. Trees with a stem diameter > 5 cm were measured using the basal stem diameter (Do)/diameter breast height (DBH, at 1.4 m height) or buttress/prop roots above 30 cm height [49]. For saplings (< 5 cm diameter), biomass and soil index were measured by sampling.

Field surveys of mangroves are usually labor-intensive and time-consuming, and regular high tides are not conducive for sampling. To avoid these complications, we conducted the sampling after low tide when water and soil environments are relatively stable [85]. The soils were profiled into different depth intervals, including 0–10 cm, 10–20 cm, 20–40 cm, 40–60 cm, 60–80 cm, and 80–100 cm (depending on the depth from surface to the underlying sand/rock layer). At each mangrove plot, three parallel samples were collected [44]. We collected soil cores at the center of each plot using an open-face PVC sediment auger (8 cm diameter), which could effectively minimize disturbance or compaction during the sampling process. Soil samples were labeled, sealed in plastic bags, and shipped to the laboratory.

### Forest structure and biomass carbon estimation

The carbon stock in mangrove ecosystems is mainly composed of above-ground biomass carbon, below-ground root biomass carbon, dead wood and litter biomass carbon, and soil sediment carbon. The proportion of dead wood and fallen litter biomass carbon reserves are usually very low and difficult to accurately collect for measurement [60]. Their biomass carbon was neglected in this study. We assumed that the carbon stock of mangrove ecosystems was mainly composed of above- and below-ground biomass carbon and soil sediment carbon.

Based on mangrove species, allometric growth equations, were used to determine the following indices of each measured vegetation: above-ground biomass (AGB), below-ground biomass (BGB), and total biomass (TBM). Common allometric equations were used if the species lack specific allometric equation [27–29, 37, 51, 52, 77, 93] (Additional file 1: Table S2). The selection of the allometric growth equation takes into account the investigation location of the equation obtained in the literature, the determination coefficient of the equation, and the rationality of the equation itself. The distances between sampling areas were maintained above 50 m in each dominant community. Compared to other biomass parts, mangrove forest litter usually domain less percentage and were not counted in this study [97].

The values of biomass were summed up for each plot and averaged to get the mean stand biomass which was then converted to Mg/ha. In our study, the mangrove forest biomass carbon stock was calculated as the vegetation biomass multiplied by a carbon conversion factor, which was 0.48 and 0.39 for the above- and below-ground portion, respectively [44].

### Determination of physical and chemical properties of mangrove soils and carbon estimation

To determine the amount of carbon stored in the soils, oil samples were dried at 60 °C using an oven until a constant weight was reached. After weighing, the soil bulk density (SBD, g/cm^3^) of each sample was determined by dividing its dry weight (g) by the given soil auger volume (cm^3^):

$${\text{SBD }}\left( {{\text{ g}}/{\text{cm}}^{3} } \right){ } = { }\left[ {{\text{dry weight }}\left( {\text{g}} \right)} \right]{ }/{ }\left[ {{\text{soil auger volume }}\left( {{\text{cm}}^{3} } \right)} \right]$$

Dry soil samples were ground using a mortar and pestle, and sieved using a 2 mm sieve to remove impurities, such as roots and shell debris. In addition, the contents of inorganic carbon (carbonate) in soil sediments were as negligible and therefore were not analyzed independently. However, carbonate was removed by treatment with diluted acid solution for samples obviously containing coral fragments [40, 85]. The carbon content concentration was analyzed using an elemental analyzer (Vario MACRO Cube, Elementar, Germany), with precision of ± 0.3%. Soil carbon stock (Mg/ha) was calculated by multiplying the soil bulk density (g/cm^3^) with the carbon content (%) and scaled by depth intervals (cm) (Additional file 1: Figure S1), and then converted according to the units [62]. The equation used to calculate carbon stock in mangrove soil was as follows:

$${\text{Carbon stock }}\left( {{\text{Mg}}/{\text{ha}}} \right) = {\text{SBD }}\left( {{\text{g}}/{\text{cm}}^{3} } \right) \times {\text{C }}\left( \% \right) \times {\text{Depth interval }}\left( {{\text{cm}}} \right)$$

### Statistical analyses

Mangrove carbon stock was calculated as the sum of all biomass (tree as the above-ground part and root as the below-ground part) and soil components. Standard errors of the total were obtained by propagating from carbon components [74]. The mean and standard deviation of all parameters were calculated in advance before analysis. The data on plant biomass, soil carbon, and other parameters were statistically analyzed by one-way analysis of variance (ANOVA). The significance of the differences between means for different mangrove forest communities was evaluated at the 95% confidence level using the Duncan’s method. We used the optimization model and Pearson’s product-moment correlation in different ways to explore the relationship between AGC and BGC variables (including linear, exponential, logarithmic, and polynomial fitting) to determine the correlation between the best fit and the best applicability [88, 89] (Additional file 1: Table S3). The relationships between the carbon component parameters and biogeographical factors were analyzed with redundancy analysis (RDA) using CANOCO 5.0 software [94]. Statistical analyses were mainly performed using SPSS (IBM 26.0). Origin (OriginLab 9.5) was used to draw graphics.

## Results

### Mangrove ecosystem carbon stock

**Fig. 2:**
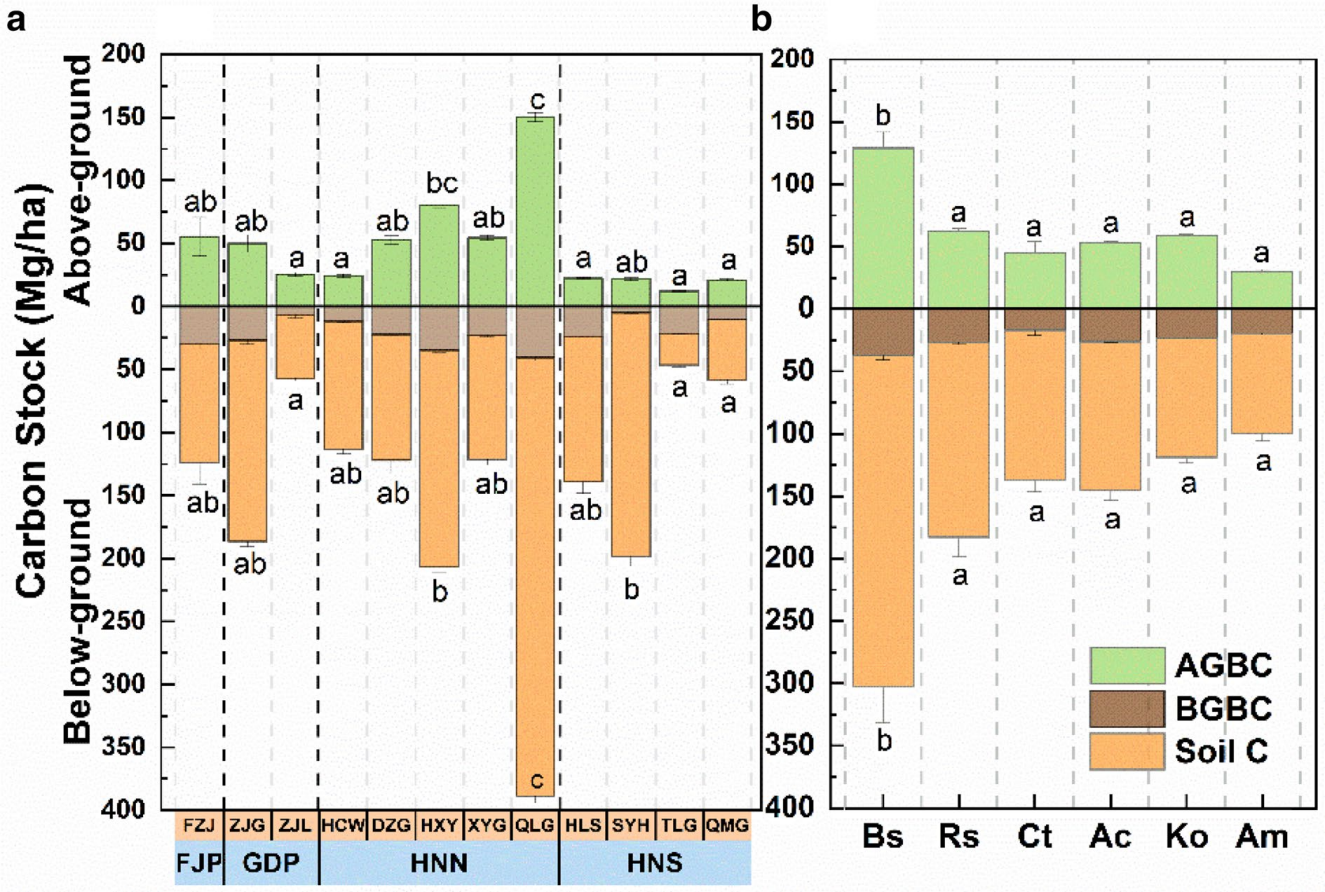
Variation of ecosystem carbon stock across different mangrove communities among different latitude regions (**a**) and dominant plant species (**b**), showing mangrove above-ground biomass carbon (AGBC), below-ground biomass carbon (BGBC), and soil carbon. Error bars represent standard deviation of biomass. Letters above histogram bars denote significant difference of above-ground carbon stock (AGC, AGC = AGBC) and below-ground carbon stock (BGC, BGC = BGBC + Soil C) among mangrove communities from multiple comparison analysis (at the *p* < 0.05 level). *B. sexangula* (Bs), *Rhizophora stylosa* (Rs), *Ceriops tagal* (Ct), *Aegiceras corniculatum* (Ac), *Kandelia obovata* (Ko), *Avicennia marina* (Am)

The variation of ecosystem carbon stocks is summarized in Fig. 2. Both above-ground carbon stock and below-ground carbon stock shared similar trends with ecosystem carbon stock. Mean ecosystem carbon stocks varied significantly among different mangrove reserves. The highest was 538.7 ± 11.3 Mg/ha in QLG, and the lowest was 58.7 ± 2.5 Mg/ha in TLG. In comparison to HNS regions, the mean mangrove carbon stock was 37.8% higher in FJP, 23.0% higher in GDP, and 102.4% higher in HNN (Fig. 2a). *B. sexangula* showed significantly higher mean carbon stock (Bs, 431.4 Mg/ha) than other species (from to 130.3 to 244.8 Mg/ha, Fig. 2b).

Among the different parts of the ecosystem's carbon stock, the soil carbon was the largest proportion, followed by above-ground biomass carbon, and the below-ground biomass carbon was the smallest. For different mangrove natural reserves, the ratio of soil carbon stock to ecosystem carbon stock ranged from 87.8% (SYH) to 42.6% (TLG), the ratio of above-ground carbon stock ranged from 30.9% (FJZ) to 9.9% (SYH). The rest was below-ground biomass carbon stock. For different dominant species, the proportion varied from 65.7% (*C. tagal*, Ct) to 53.5% (*K. obovata*, Ko), and the proportion of above-ground biomass carbon ranged from 33.1% (*K. obovata*, Ko) to 23.0% (*A. marina*, Am).

### Forests structure

Total biomass (TBM) was the lowest in SYH (58.1 ± 25.0 Mg/ha) and significantly the highest in QLG (538.7 ± 11.3 Mg/ha) among all survey sites. The means of average tree height (ATH), canopy density (CD), and tree density (TD) showed significant differences between mangrove reserves. The highest average tree height was 5.8 ± 0.8 m in ZJL, and the highest tree density was ZJL (2.4 ± 0.5 unit/m^2^) (Additional file 1: Figure S1). For mangrove floristic structure, *B. sexangula* (Bs) showed higher TBM (36.5 ± 19.3 Mg/ha), diameter breast height (DBH) (24.3 ± 10.8 cm), ATH (4.7 ± 1.2 m), and CD (0.9 ± 0.02%) than other species.

### Relationship between mangrove above- and below-ground carbon stock

**Fig. 3:**
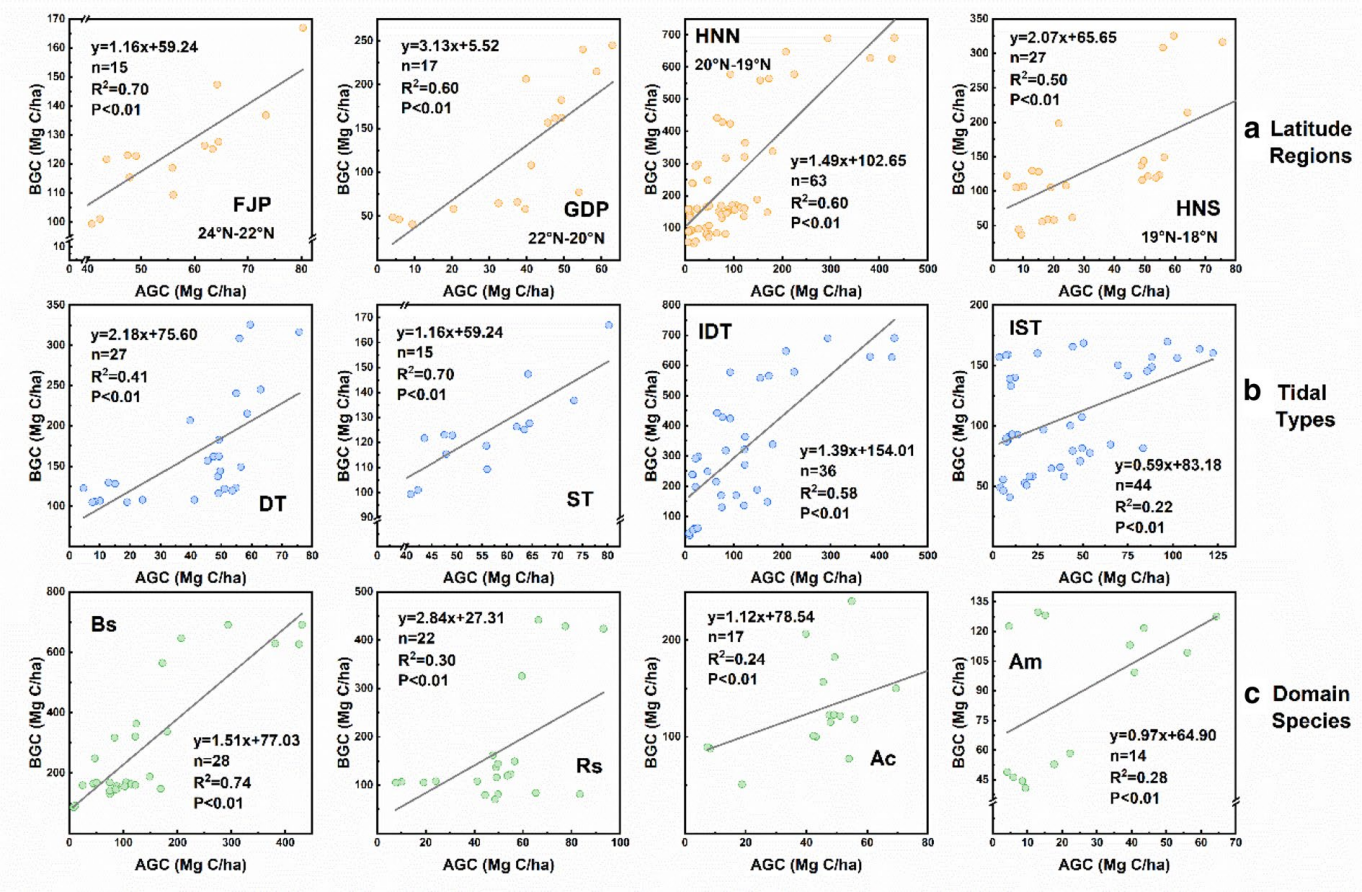
Correlation pattern of mangrove above-ground carbon (AGC) and below-ground carbon (BGC) affected by biogeographic factors: **a** latitude region, **b** tidal type, and **c** domain species. Tidal types: diurnal tide (DT), semidiurnal tide (ST), mixed tide (irregular diurnal tide IDT, irregular semidiurnal tide IST)

Above-ground carbon (AGC) and below-ground carbon (BGC) stock of the different mangrove communities are shown in Fig. 3. AGC and BGC present a significant linear correlation (p < 0.01), and correlations between AGC and BGC vary under different research perspectives, especially under tidal types and domain species. But, the overall correlation shows a significant positive rise (p < 0.01).

The variation of AGC (3.66–430.98 Mg/ha) and BGC (50.86–690.98 Mg/ha) was greater in HNN among latitude regions, and FJP (R^2^ = 0.70, p < 0.01) had a better regression coefficient than either GDP (R^2^ = 0.60, p < 0.01), HNN (R^2^ = 0.60, p < 0.01) and HNS (R^2^ = 0.50, p < 0.01) (Fig. 3a). This trend was also seen within the scope of different tidal types and domain species communities, whereas more variation and better regression existed in IDT (R^2^ = 0.60, p < 0.01) and Bs (R^2^ = 0.60, p < 0.01) (Fig. 3bc).

### Effects of structural variables on the relationship between AGC and BGC

**Fig. 4:**
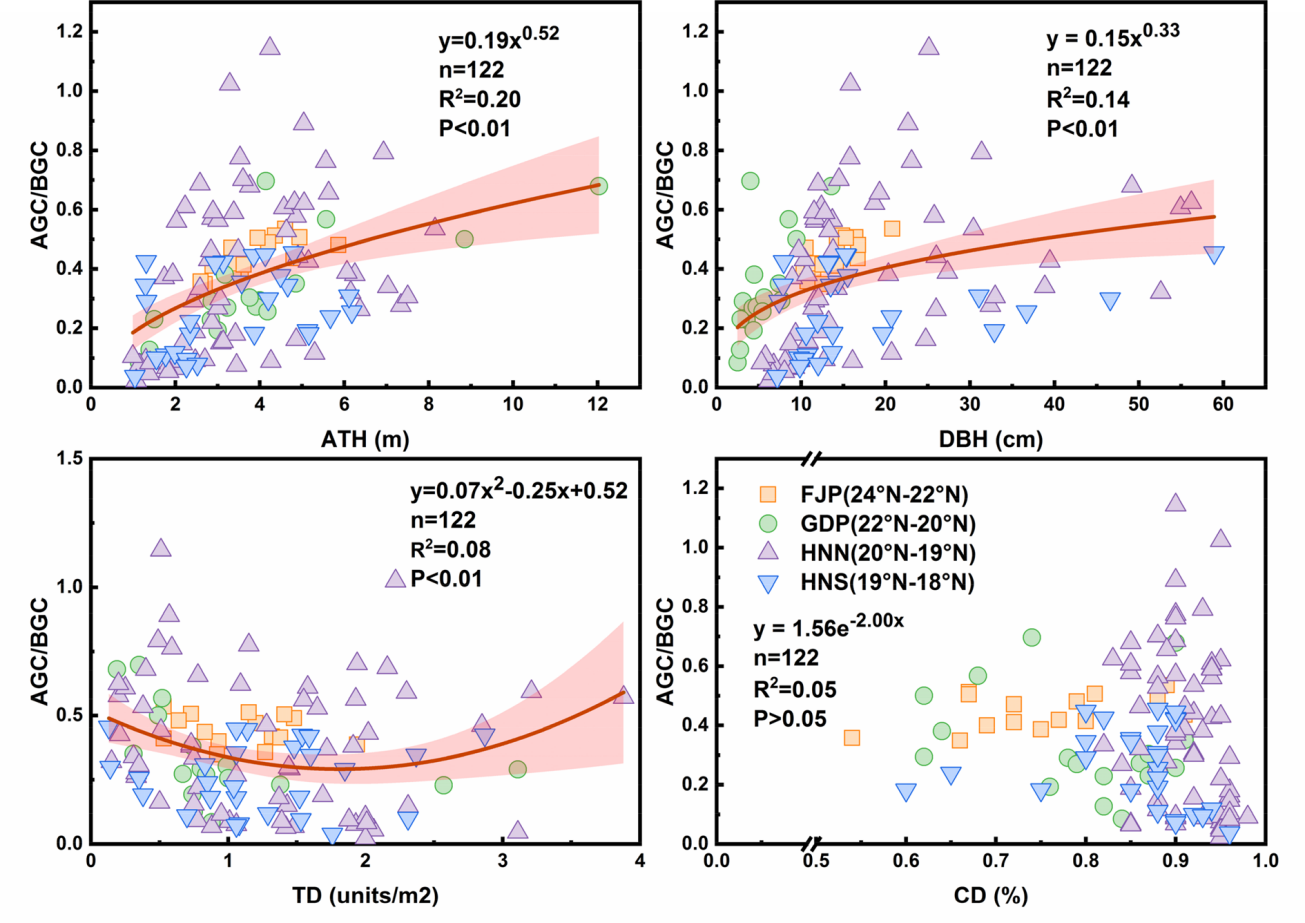
The effect of structural variables on the relationship between AGC and BGC: **a** average tree height (ATH, m), **b** diameter breast height (DBH, cm), **c** tree density (TD, units/m^2^), **d** canopy density (CD, %). Circles, squares, and triangles indicate different mangrove latitude regions. Red shaded areas indicate a 95% confidence interval for the fitted line

The relationships between AGC/BGC and forest structure factors for different mangrove communities are shown in Fig. 4. A correlation analysis indicated that AGC/BGC was significantly (p < 0.01) correlated with average tree height (ATH), diameter breast height (DBH), and tree density (TD). The AGC/BGC tended to increase gradually with increasing ATH (R^2^ = 0.20, p < 0.001) and DBH (R^2^ = 0.14, p < 0.001), fitting a power equation (Fig. 4a, b). This was also true for the relationship between AGC/BGC and TD, but the correlation (R^2^ = 0.08, p < 0.001) fitted a polynomial equation (Fig. 4c). No significant correlation was found between AGC/BGC and CD. Overall, the relationships between AGC/BGC and forest structural factors showed their own characteristics for each latitude area. Compared with other latitude regions, the AGC/BGC in HNN changed and was widely distributed with the ATH, DBH, and TD. At other latitudes, the correlation distribution was relatively concentrated.

## Discussion

### Mangrove forest carbon stock distribution

In forests, soil and vegetation biomass are the main components of ecosystem carbon stock, and their proportional changes reflect the interaction between the components of the carbon stock [24]. In this study, we found that the average above-ground carbon stock in each region was sizeable (ranging from 12.0 to 150.2 Mg/ha), but average below-ground carbon stock was dominant (ranging from 46.6 to 388.6 Mg/ha), accounting for 69–91% of total carbon stock at the sites studied in China. The carbon stock ratio of soil to ecosystem was 0.43–0.88 for mangroves in China; the same trend also exists when expanded to global mangroves (Fig. 2, Additional file 1: Table S1). The results showed that the below-ground carbon stock was the dominant component of entire mangrove carbon stock. This might be due to the fact that mangroves have developed adaptive root structures to endure marine tidal floods and anaerobic conditions [48]. Anaerobic conditions, abundant sunshine, high salinity, and high sulfate concentrations significantly reduced carbon loss from below-ground portions via soil respiration and carbon emission, thereby increasing the distribution of rich carbon in the below-ground portion and in sediments. [14, 106]. In addition to high productivity and litter accumulation, mangrove forests can also trap large quantities of river debris in their sediments due to their special and complex root systems [41, 67]. The unique habitats and plant ecophysiological adaptations of mangrove forests give them a distinct carbon storage pattern and distinguish them from other forest ecosystems in the same region.

In the global ecosystem, the total soil carbon is far greater than the sum of carbon in vegetation and atmosphere [43]. Soil carbon plays a key role in mangrove carbon stock and recycling [33, 96]. As one of the most important carbon sinks in global ecosystems, mangroves are the best choice to reduce the impact of climate change by accurately assessing and protecting mangrove ecosystem carbon stock, especially the below-ground soil [87].

The application and improvement of remote sensing technology and allometric growth models facilitate estimates of the carbon pool in above-ground mangrove vegetation. However, the estimation of below-ground carbon, especially soil carbon stocks, is still limited due to its unclear relationship with the above-ground carbon, difficulty in sampling, and insufficient data. Since below-ground carbon stocks are the dominant components of mangrove carbon stocks, a better understanding of the connections between mangrove carbon components is necessary for accurately estimating below-ground/total carbon stocks. Blue carbon management and ecosystem service evaluations need more accurate estimates of ecosystem mangrove carbon stocks [68].

### Relationship between BGC and AGC for mangrove forests

**Fig. 5:**
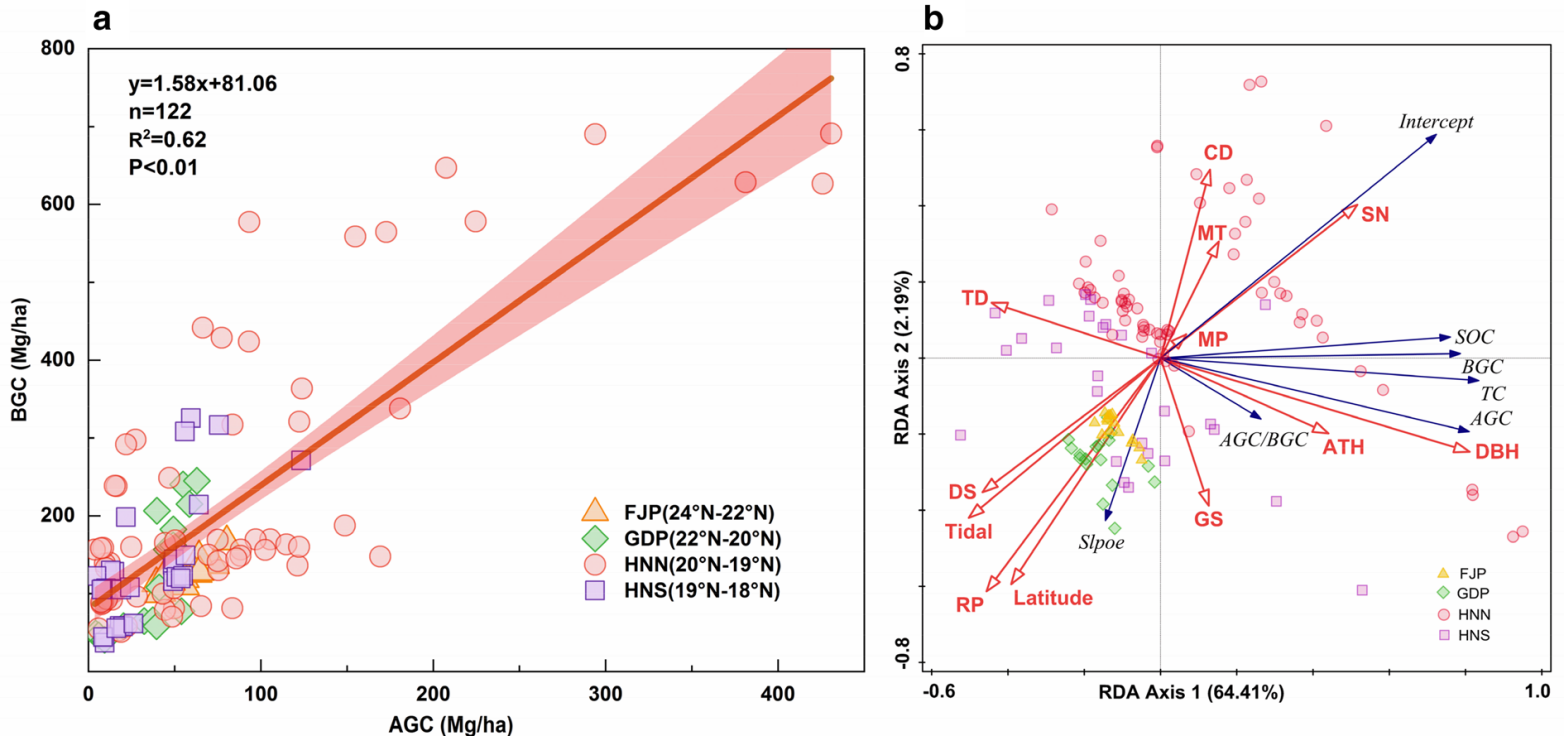
**a** Entire relationship between AGC and BGC in the mangrove ecosystem across different mangrove communities in China. Circles, squares, and triangles indicate different mangrove latitude regions. Red shaded areas indicate a 95% confidence interval for the fitted line. **b** Redundancy analysis (RDA) diagram for the correlation between carbon stock component characteristics (blue line) and structural, biogeographical variables (red line). Note: The slope and intercept are the parameters of the linear correlation between AGC and BGC; Reserve position (RP), Domain species (DS), Domain species numbers (SN), Mean annual temperature (MT), Mean annual precipitation (MP), Geomorphic settings (GS, types include: lagoon, estuary, and open coast)

Through a variety of comparative analyses, we found strong positive linear relationships between the above- and below-ground carbon stocks in mangroves (Figs. 3, 5, Additional file 1: Table S3), which slightly varied between different locations, latitudes, and species. At the latitudinal scale of the protected area, the above-ground and below-ground carbon stocks (mangrove below-ground biomass and soil carbon) of mangroves showed good correlations. We found a similar relationship between AGC and BGC in the range of different site scales, and in most mangrove species (except for Rs, Ac, and Ct, where no significant correlation was found) (Figs. 3, 5, Additional file 1: Table S3).

The relationship between mangrove above- and below-ground carbon stocks is inseparable from the characteristics and mechanisms of mangrove ecosystem carbon cycles. Mangroves rely on the photosynthesis of vegetation to import heterogeneous carbon from the atmosphere to increase carbon stock. The carbon produced by vegetation is distributed and transferred proportionally to below-ground and other carbon stocks through root transportation, litter decomposition, and dead wood storage [2]. Ultimately, most of the carbon is generated in situ and stored below-ground, eventually becoming sedimentary carbon [53, 72, 80]. Although mangroves also capture carbon from upstream rivers or tidal water (laden with fine particles from adjacent coastal oceans), mangrove vegetation (distribution, size, shape, and distribution pattern) plays a decisive role in the deposition process [65, 66]. Similarly, characterization of soil stable carbon isotopes indicated that mangrove vegetation was an important, direct or indirect, source of soil carbon stock [5, 7, 55]. Our findings provide useful ideas and perspectives for better estimating carbon stock in the mangrove ecosystem, but more accurate data and further exploration are needed to determine whether these findings are universally applicable.

### Factors affecting the relationship between BGC and AGC in mangrove forests

We found a correlation between mangrove AGC and BGC with the degree of this correlation varying according to different habitats of the mangrove ecosystem at different scales (Fig. 3). This correlation was strongly affected by latitude, tide, climatic characteristics, dominant species, and the number of total groups (Fig. 5).

The effects of biogeographic and floristic factors on the components of mangrove carbon stock and their relationships are multi-level. For the ratio of AGC to BGC, the influences of stand structural characteristics (DBH, ATH, TD), and geomorphic settings are the most important. The tide, peculiar to mangroves, promotes nutrient exchange and aeration in the soil layer, thereby reducing the accumulation of sulfur compounds, and ultimately controlling the decomposition rate of organic matters in the mangrove soils. Regular and continuous tidal flooding promotes the mineralization process and the accumulation of terrestrial soil carbon [18, 61]. The tide also carries a large amount of sediments, which promote the lateral capture of mud due to the reduced water flow in mangroves [99].

Carbon accumulation of trees increases with tree size and 70% of the biomass change is determined by large trees [91]. This partially explains the variation of tree density in HNN and HNS mangrove carbon stock (Fig. 3) and the effect of tree density on AGC/BGC (Fig. 4). Similarly, the structural characteristics of mangrove vegetation indirectly regulate the distribution and relationship of mangrove carbon stock (Fig. 5), through sediment accumulation, biomass changes, and biogeochemical variables [91]. These factors greatly impact the distribution of carbon stock in the above- and below-ground biomass of mangrove forests and the relationships between AGC and BGC.

The large spatial pattern of mangrove carbon stock is largely controlled by climatic factors, such as annual precipitation, mean temperature, and frequency of tropical cyclone landfall [88]. In addition to climatic factors, the spatial pattern (biogeographical factors) of ecosystem carbon storage is usually controlled by the local water environment [19, 25, 42, 92]. By affecting the deposition rate, the type and species of vegetation (floristic factors) also affect the carbon stock in mangroves [103]. The amount and dynamics of carbon stocks vary considerably in different mangrove soils and roots, based on the effects of tidal gradient, vegetation biomass and productivity, species composition, and suspended matter deposition [18, 38].

**Fig. 6:**
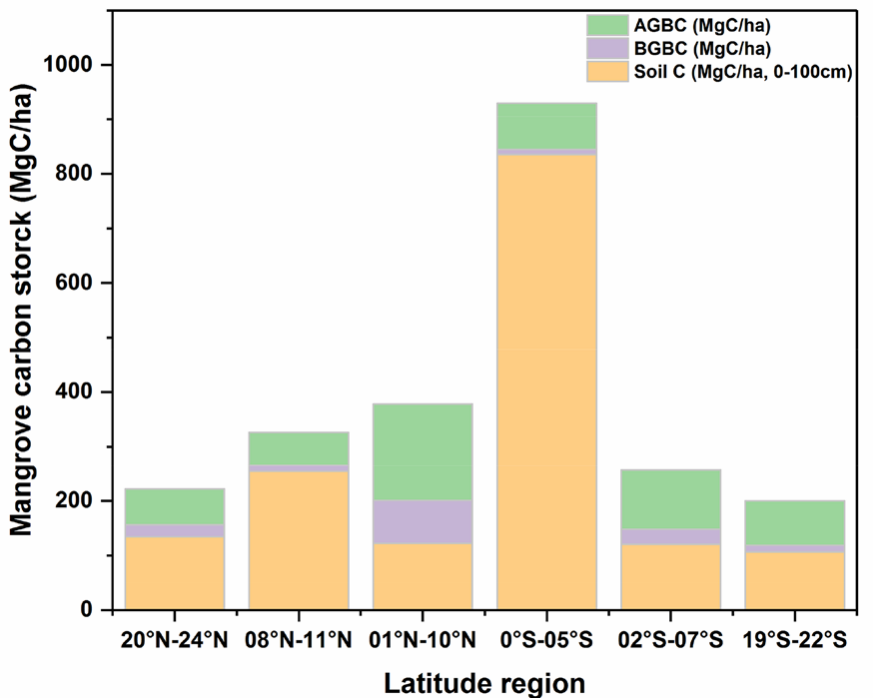
Carbon stock of mangrove ecosystems in different latitudes. Different colors indicate Above-ground biomass carbon (AGBC), Below-ground biomass carbon (BGBC) and Soil C (0–100 cm depth). Data from Additional file 1: Table S1

**Fig. 7:**
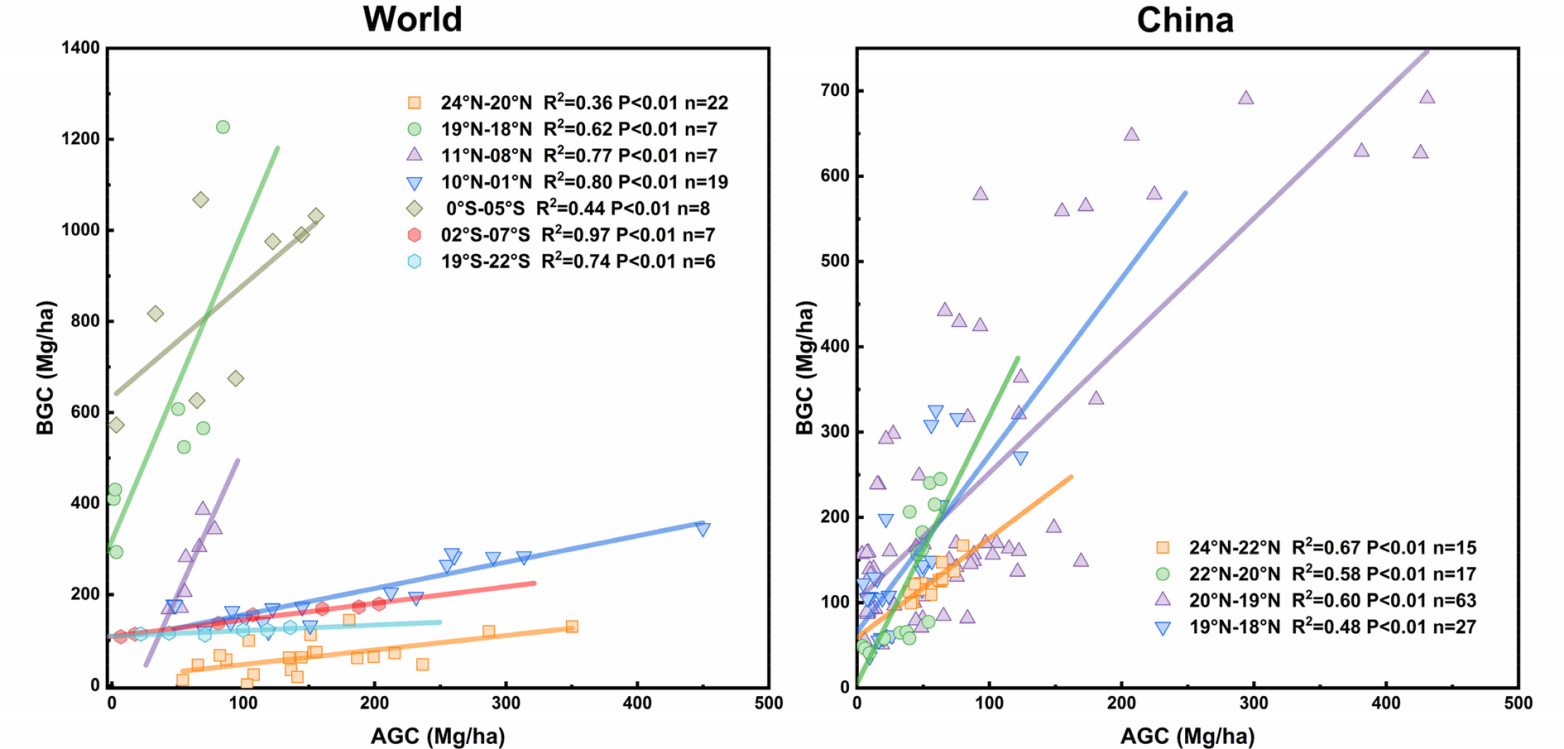
Relationship between above-ground carbon stock (AGC, AGC = AGBC) and below-ground carbon stock (BGC, BGC = BGBC + Soil C) of mangrove ecosystems across different mangrove communities around the world and in China. Lines represent linear regressions. Data from Fig. 2 and Additional file 1: Table S1

In mangrove ecosystems, AGC and BGC correlated well at different latitudes on a global scale, except that correlations in the range of 0° S–05° S was not significant. This might be related to the excessively high soil carbon stock of mangroves in the range of 0° S–05° S and the cutting of above-ground vegetation (Fig. 6). Except for 08° N–11° N, in the other latitude ranges, the slopes of the correlations between AGC and BGC in mangrove ecosystems showed a latitudinal pattern, and gradually increased with latitude (Fig. 7).

As a result, the relationship between the mangrove above- and below-ground carbon stocks that we found at different scales can make up for the lack of understanding in mangrove carbon stock partition. In particular, we took the mangrove below-ground biomass carbon and soil carbon as an integral part to conduct a comparative study with the above-ground biomass carbon. It will refresh people with a unique research perspective for a more comprehensive understanding of mangrove carbon stock pattern and carbon cycle process. It provides a foundation for further improving representativeness and accuracy of the relationships between above- and below-ground carbon stocks, and exploring more applicable relationships on a broader scale. At the same time, accurate relationships between above- and below-ground carbon stocks can also serve as a bridge between remote sensing biomass estimation and traditional sample plot surveys, amplifying the long-term and spatial-scale advantages of remote sensing estimation and the comprehensiveness/accuracy of field carbon stock estimation (especially the below-ground part). It provides useful help for the improvement of methods for monitoring and estimation of mangrove carbon stock distribution.

At different latitudes and changes in the study scale range, the latitudinal distribution pattern of terrestrial forest carbon stock weakens as the scale decreases [98]. Differences in below-ground carbon stock, especially the dominant soil carbon stock, result from differences in dominant species, soil depth, soil bulk density, and protected areas [36]. These factors may explain some regular variations present in this study (Fig. 7), and should be further considered in future estimations and studies on changes of mangrove carbon stock. The high carbon density of mangrove ecosystems (especially the soil) is affected by a wide range of external factors, such as primary productivity, geographic location, species composition, and protected status [1, 86]. The organic carbon of mangrove forest soil may extend several meters in depth, though carbon stock is concentrated in the top meter of soil [33]. We may have underestimated soil carbon considering our data was based on the upper 100 cm of soil [35, 50]. Therefore, the actual carbon stock of mangrove ecosystems may exceed the estimation of this study. This perspective highlights the importance of accurate estimation at different levels and scales, especially when applying correlations between AGC and BGC to estimate mangrove carbon stock and storage using remote sensing techniques.

### Implications for mangrove carbon restoration and management

Monitoring carbon stocks at different scales enables better understanding and protection of mangrove ecosystems. From a global scale, the highest carbon stock in mangrove ecosystems occurs at tropical latitudes and decreases with increasing latitude, due to latitudinal differences in climate conditions [16, 17, 51, 104]. Therefore, at different regional scales, forest stand composition and structural changes of mangroves usually lead to different soil carbon distributions [38, 57], which accounts for the diversity of our results.

On the southeast coast of China, mangroves have been severely disturbed by nearby residential areas and aquaculture, with extremely cold temperature, disease and pest outbreaks, biological invasions, and high anthropogenic stresses, such as pollutants [23, 64]. The state of mangroves varies greatly due to differences in priority level, time to establish reserves in different regions, as well as changes in local forest management capabilities (Table 1).

Extensive and accurate dynamic monitoring of mangrove carbon storage is limited since mangroves distributes along a long coastline but in scattered areas, as well as the ability to apply remote sensing technology is also insufficient [56, 84, 95]. Our findings can effectively compensate for these limitations. Large-scale accurate estimation of carbon stock for mangrove ecosystems can be achieved by applying the local relationship between above- and below-ground ecosystem carbon stocks after remote sensing. These findings will support efforts to monitor mangrove ecosystem carbon cycles and provide a basis for the development of sustainable management programs for coastal blue carbon and mangrove forests.

## Conclusions

We found positive relationships between above- and below-ground carbon stocks in mangrove forests with different properties and over different spatial scales, suggesting that below-ground and total mangrove carbon stocks can be estimated based on above-ground carbon stocks. The correlation coefficients for such relationships differ significantly among different spatial scales (from a forest stand, to a region, to globally) and different community characteristics. In order to apply these relationships to mangrove carbon stock estimates, the appropriate AGC and BGC relationship must be carefully determined for the scale, with consideration of the characteristics of mangrove biogeographic environments and forest stand structure. Our findings provide a reasonable scientific foundation for estimation of mangrove ecosystem carbon stocks by taking advantages of other technologies, including remote sensing. Our findings will ultimately enable more accurate evaluation of the role of mangrove protection in global carbon cycle.

## Supplementary Information

**Additional file 1: Figure S1.** Different mangrove forests vegetation and soil properties. **Table S1.** Carbon stocks of mangrove ecosystems in different parts of the world. **Table S2.** Allometric equations for various mangrove based on DBH. **Table S3.** The main results of AGC and BGC relation fitting by using the optimization model.

## Data Availability

The mean annual precipitation, and geomorphic settings are available from China Meteorological Data Service Center (http://data.cma.cn). Part of data are available for Additional file information reference.

## Abbreviations

GEP: Gross ecosystem production
AGB: Above-ground biomass
BGB: Below-ground biomass
TBM: Total biomass
SBD: Soil bulk density
AGC: Above-ground carbon stock
BGC: Below-ground carbon stock
ATH: Average tree height
DBH: Diameter breast height
TD: Tree density
CD: Canopy density
RP: Reserve position
DS: Domain species
SN: Species numbers
MT: Mean annual temperature
MP: Mean annual precipitation
GS: Geomorphic settings
DT: Diurnal tide
ST: Semidiurnal tide
IDT: Irregular diurnal tide
IST: Irregular semidiurnal tide
