# Supplementary material for: Relationships between above- and below-ground carbon stocks in mangrove forests facilitate better estimation of total mangrove blue carbon
Source: Carbon Balance Manag. 2021 Mar 17;16:8. doi: 10.1186/s13021-021-00172-9 (PMC7972349; doi:10.1186/s13021-021-00172-9)
Supplement: Supplementary file 1 — Additional file 1: Figure S1. Different mangrove forests vegetation and soil properties. Table S1. Carbon stocks of mangrove ecosystems in different parts of the world. Table S2. Allometric equations for various mangrove based on DBH. Table S3. The main results of AGC and BGC relation fitting by using the optimization model. [file 13021_2021_172_MOESM1_ESM.docx]

***Revised Manuscript for Carbon Balance and Management***

**Additional Material**

**Relationships between above- and below-ground carbon stocks in mangrove forests facilitate better estimation of total mangrove blue carbon**

Yuchen Meng^1,2^, Jiankun Bai^1,2^, Ruikun Gou^1,2^, Xiaowei Cui^1,4^, Jianxiang Feng^3^, Zheng Dai^2^, Xiaoping Diao^5,6^, Xiaoshan Zhu^2*^, Guanghui Lin^1,2^*

^1^Key Laboratory for Earth System Modeling, Ministry of Education, Department of Earth System Science, Tsinghua University, Beijing, 100084, China

^2^Institute of Ocean Engineering, Tsinghua Shenzhen International Graduate School, Shenzhen, 518055 Guangdong, China

^3^School of Marine Sciences, Sun Yat-sen University, Zhuhai, 510275 Guangdong, China

^4^Kunming Institute of Survey and Design, State Forestry and Grassland Administration, Kunming, 650216 Yunnan, China

^5^College of Life Science, Hainan Normal University, Haikou, 571158 Hainan, China

^6^State Key Laboratory of South China Sea Marine Resource Utilization, Hainan University, Haikou, 570228 Hainan, China

***Correspondence**: E-mail: GH Lin (lingh@tsinghua.edu.cn or lin.guanghui@sz.tsinghua.edu.cn); XS Zhu (zhu.xs@sz.tsinghua.edu.cn)

**Address**: Tsinghua University, Beijing, 100084, China





**Figure S1** Different mangrove forests vegetation and soil properties. **a):** vegetation properties (including TD, SN, DBH, CD and ATH) and **b)** to **d):** soil properties (including C (%), SWC (%) and BD (g/cm^3^)) distribution. Red open circles indicate the observed values.

**Table S1.** Carbon stocks of mangrove ecosystems in different parts of the world

| **Positions** | **Country/**  **Region** | **Latitude range** | **AGBC (MgC/ha)** | **BGBC (MgC/ha)** | **Soil C (MgC/ha, 0-100cm)** | **ABC (MgC/ha)** | **BGC (MgC/ha)** | **Reference** |
| --- | --- | --- | --- | --- | --- | --- | --- | --- |
| Futian mangrove nature reserve, Shenzhen bay | China | 20°N-24°N | 24.2 | 3.7 | 104.2 | 24.2 | 107.9 | (Peng et al. 2016) |
| Futian mangrove nature reserve, Shenzhen bay | China | 20°N-24°N | 99.0 | 40.5 | 63.7 | 99.0 | 104.2 | (Peng et al. 2016) |
| Futian mangrove nature reserve, Shenzhen bay | China | 20°N-24°N | 45.4 | 15.2 | 50.2 | 45.4 | 65.4 | (Peng et al. 2016) |
| Futian mangrove nature reserve, Shenzhen bay | China | 20°N-24°N | 57.0 | 16.5 | 70.6 | 57.0 | 87.0 | (Peng et al. 2016) |
| Futian mangrove nature reserve, Shenzhen bay | China | 20°N-24°N | 12.4 | 3.0 | 51.0 | 12.4 | 53.9 | (Peng et al. 2016) |
| Futian mangrove nature reserve, Shenzhen bay | China | 20°N-24°N | 66.5 | 18.1 | 64.1 | 66.5 | 82.2 | (Peng et al. 2016) |
| Futian mangrove nature reserve, Shenzhen bay | China | 20°N-24°N | 34.6 | 16.8 | 119.6 | 34.6 | 136.4 | (Lunstrum and Chen 2014) |
| Futian mangrove nature reserve, Shenzhen bay | China | 20°N-24°N | 2.2 | 0.6 | 102.3 | 2.2 | 102.9 | (Lunstrum and Chen 2014) |
| Futian mangrove nature reserve, Shenzhen bay | China | 20°N-24°N | 60.5 | 16.4 | 170.4 | 60.5 | 186.8 | (Lunstrum and Chen 2014) |
| Futian mangrove nature reserve, Shenzhen bay | China | 20°N-24°N | 61.9 | 11.3 | 123.6 | 61.9 | 134.9 | (Lunstrum and Chen 2014) |
| Ganges-Brahmaputra Delta | Bangladesh | 20°N-24°N | 62.1 | 27.1 | 117.2 | 62.1 | 144.3 | (Donato et al. 2011) |
| Ganges-Brahmaputra Delta | Bangladesh | 20°N-24°N | 111.2 | 45.1 | 106.2 | 111.2 | 151.2 | (Donato et al. 2011) |
| Qinzhou bay | China | 20°N-24°N | 144.4 | 29.8 | 150.8 | 144.4 | 180.7 | (He et al. 2017) |
| Qinzhou bay | China | 20°N-24°N | 73.8 | 20.9 | 132.6 | 73.8 | 153.5 | (He et al. 2017) |
| Qinzhou bay | China | 20°N-24°N | 63.3 | 26.8 | 172.4 | 63.3 | 199.2 | (He et al. 2017) |
| Zhanjiang Nature Reserve, Yingluo Bay | China | 20°N-24°N | 129.9 | 44.7 | 305.7 | 129.9 | 350.4 | (Wang et al. 2013) |
| Zhanjiang Nature Reserve, Yingluo Bay | China | 20°N-24°N | 71.4 | 22.3 | 192.9 | 71.4 | 215.2 | (Wang et al. 2013) |
| Zhanjiang Nature Reserve, Yingluo Bay | China | 20°N-24°N | 46.6 | 15.2 | 221.3 | 46.6 | 236.5 | (Wang et al. 2013) |
| Zhanjiang Nature Reserve, Yingluo Bay | China | 20°N-24°N | 73.6 | 11.0 | 144.7 | 73.6 | 155.7 | (Wang et al. 2013) |
| Zhanjiang Nature Reserve, Yingluo Bay | China | 20°N-24°N | 19.2 | 7.9 | 133.4 | 19.2 | 141.3 | (Wang et al. 2013) |
| Dongzhai harbor | China | 20°N-24°N | 119.3 | 67.0 | 220.0 | 119.3 | 287.0 | (Liu et al. 2014) |
| Xamach | Mexico | 18°N-19°N | 1.4 | 3.4 | 407.0 | 1.4 | 410.4 | (Adame et al. 2013) |
| La Raya | Mexico | 18°N-19°N | 3.4 | 7.4 | 286.0 | 3.4 | 293.4 | (Adame et al. 2013) |
| El Playon | Mexico | 18°N-19°N | 2.5 | 4.8 | 426.0 | 2.5 | 430.8 | (Adame et al. 2013) |
| Laguna Negra | Mexico | 18°N-19°N | 54.8 | 27.9 | 496.0 | 54.8 | 523.9 | (Adame et al. 2013) |
| Cayo Culebra | Mexico | 18°N-19°N | 69.6 | 57.4 | 508.0 | 69.6 | 565.4 | (Adame et al. 2013) |
| Hualaxtoc | Mexico | 18°N-19°N | 50.4 | 30.4 | 577.0 | 50.4 | 607.4 | (Adame et al. 2013) |
| Isla Pitaya | Mexico | 18°N-19°N | 84.6 | 61.1 | 1166.0 | 84.6 | 1227.1 | (Adame et al. 2013) |
| Kien Vang Protection Forest (KVPF) | Vietnam | 08°N-11°N | 69.2 | 7.7 | 378.3 | 69.2 | 386.0 | (Nam et al. 2016) |
| Can Gio Mangrove Biosphere Reserve (CGMBR) | Vietnam | 08°N-11°N | 56.1 | 9.7 | 272.3 | 56.1 | 282.0 | (Nam et al. 2016) |
| Can Gio Mangrove Forest Park (CGM) | Vietnam | 08°N-11°N | 43.3 | 17.5 | 150.2 | 43.3 | 167.7 | (Dung et al. 2016) |
| Can Gio Mangrove Forest Park (CGM) | Vietnam | 08°N-11°N | 52.4 | 10.6 | 159.9 | 52.4 | 170.5 | (Dung et al. 2016) |
| Can Gio Mangrove Forest Park (CGM) | Vietnam | 08°N-11°N | 55.3 | 12.5 | 193.4 | 55.3 | 205.9 | (Dung et al. 2016) |
| Can Gio Mangrove Biosphere Reserve (CGMBR) | Vietnam | 08°N-11°N | 78.3 | 10.4 | 333.0 | 78.3 | 343.4 | (Nam et al. 2016) |
| Kien Vang Protection Forest (KVPF) | Vietnam | 08°N-11°N | 66.5 | 11.1 | 293.2 | 66.5 | 304.3 | (Nam et al. 2016) |
| Yap | Indo-Pacific | 01°N-10°N | 144.8 | 67.6 | 105.2 | 144.8 | 172.7 | (Donato et al. 2011) |
| Yap | Indo-Pacific | 01°N-10°N | 449.8 | 221.1 | 125.2 | 449.8 | 346.3 | (Donato et al. 2011) |
| Yap | Indo-Pacific | 01°N-10°N | 290.2 | 160.0 | 123.2 | 290.2 | 283.1 | (Donato et al. 2011) |
| Yap | Indo-Pacific | 01°N-10°N | 255.0 | 141.5 | 124.2 | 255.0 | 265.6 | (Donato et al. 2011) |
| Yap | Indo-Pacific | 01°N-10°N | 212.4 | 90.6 | 114.2 | 212.4 | 204.8 | (Donato et al. 2011) |
| Palau | Indo-Pacific | 01°N-10°N | 122.3 | 57.0 | 113.2 | 122.3 | 170.1 | (Donato et al. 2011) |
| Kosrae | Indo-Pacific | 01°N-10°N | 260.6 | 164.7 | 119.2 | 260.6 | 283.8 | (Donato et al. 2011) |
| Kosrae | Indo-Pacific | 01°N-10°N | 313.7 | 163.1 | 121.2 | 313.7 | 284.2 | (Donato et al. 2011) |
| Kosrae | Indo-Pacific | 01°N-10°N | 231.5 | 72.9 | 122.2 | 231.5 | 195.0 | (Donato et al. 2011) |
| Kosrae | Indo-Pacific | 01°N-10°N | 258.7 | 171.2 | 120.2 | 258.7 | 291.4 | (Donato et al. 2011) |
| Wori , North Sulawesi | Indonesia | 01°N-10°N | 46.9 | 15.5 | 163.0 | 46.9 | 178.5 | (Donato et al. 2011) |
| Wori , North Sulawesi | Indonesia | 01°N-10°N | 48.2 | 15.1 | 162.0 | 48.2 | 177.1 | (Donato et al. 2011) |
| Wori , North Sulawesi | Indonesia | 01°N-10°N | 91.6 | 28.1 | 136.0 | 91.6 | 164.1 | (Donato et al. 2011) |
| Sulawesi | Indo-Pacific | 01°N-10°N | 150.8 | 23.1 | 109.2 | 150.8 | 132.3 | (Donato et al. 2011) |
| Sulawesi | Indo-Pacific | 01°N-10°N | 70.3 | 8.8 | 111.2 | 70.3 | 120.0 | (Donato et al. 2011) |
| Sulawesi | Indo-Pacific | 01°N-10°N | 119.1 | 8.2 | 110.2 | 119.1 | 118.4 | (Donato et al. 2011) |
| Sulawesi | Indo-Pacific | 01°N-10°N | 90.4 | 26.5 | 112.2 | 90.4 | 138.6 | (Donato et al. 2011) |
| Sulawesi | Indo-Pacific | 01°N-10°N | 114.8 | 23.7 | 116.2 | 114.8 | 139.9 | (Donato et al. 2011) |
| Sulawesi | Indo-Pacific | 01°N-10°N | 101.2 | 31.0 | 115.2 | 101.2 | 146.2 | (Donato et al. 2011) |
| Bunaken | Indonesia | 0°S-05°S | 122.4 | 10.6 | 965.1 | 122.4 | 975.7 | (Murdiyarso et al. 2015) |
| Kubu Raya | Indonesia | 0°S-05°S | 3.3 | 1.0 | 571.6 | 3.3 | 572.6 | (Murdiyarso et al. 2015) |
| Teminabuan | Indonesia | 0°S-05°S | 94.2 | 14.3 | 660.5 | 94.2 | 674.8 | (Murdiyarso et al. 2015) |
| Tanjung Puting | Indonesia | 0°S-05°S | 64.7 | 5.6 | 620.9 | 64.7 | 626.5 | (Murdiyarso et al. 2015) |
| Bintuni | Indonesia | 0°S-05°S | 33.2 | 5.8 | 811.6 | 33.2 | 817.4 | (Murdiyarso et al. 2015) |
| Sembilang | Indonesia | 0°S-05°S | 144.2 | 10.9 | 979.5 | 144.2 | 990.4 | (Murdiyarso et al. 2015) |
| Timika | Indonesia | 0°S-05°S | 155.3 | 17.0 | 1014.8 | 155.3 | 1031.8 | (Murdiyarso et al. 2015) |
| Cilacap | Indonesia | 0°S-05°S | 67.6 | 8.3 | 1059.2 | 67.6 | 1067.5 | (Murdiyarso et al. 2015) |
| Java | Indo-Pacific | 02°S-07°S | 17.6 | 5.2 | 108.2 | 17.6 | 113.4 | (Donato et al. 2011) |
| Java | Indo-Pacific | 02°S-07°S | 6.7 | 0.4 | 107.2 | 6.7 | 107.6 | (Donato et al. 2011) |
| Borneo | Indo-Pacific | 02°S-07°S | 107.4 | 27.6 | 128.2 | 107.4 | 155.7 | (Donato et al. 2011) |
| Borneo | Indo-Pacific | 02°S-07°S | 188.1 | 46.9 | 126.2 | 188.1 | 173.1 | (Donato et al. 2011) |
| Borneo | Indo-Pacific | 02°S-07°S | 203.4 | 52.0 | 127.2 | 203.4 | 179.2 | (Donato et al. 2011) |
| Borneo | Indo-Pacific | 02°S-07°S | 160.1 | 40.0 | 129.2 | 160.1 | 169.2 | (Donato et al. 2011) |
| Borneo | Indo-Pacific | 02°S-07°S | 81.0 | 18.8 | 118.2 | 81.0 | 137.0 | (Donato et al. 2011) |
| Dampier | Australia | 19°S-22°S | 22.0 | 8.3 | 105.2 | 22.0 | 113.4 | (Alongi et al. 2000) |
| Dampier | Australia | 19°S-22°S | 118.4 | 17.5 | 104.2 | 118.4 | 121.6 | (Alongi et al. 2000) |
| Port Hedland | Australia | 19°S-22°S | 99.8 | 14.2 | 108.2 | 99.8 | 122.3 | (Alongi et al. 2000) |
| Port Hedland | Australia | 19°S-22°S | 70.8 | 4.5 | 107.2 | 70.8 | 111.6 | (Alongi et al. 2000) |
| Port Hedland | Australia | 19°S-22°S | 135.7 | 21.8 | 106.2 | 135.7 | 127.9 | (Alongi et al. 2000) |
| Mangrove Bay | Australia | 19°S-22°S | 43.4 | 6.3 | 109.2 | 43.4 | 115.4 | (Alongi et al. 2000) |

**Notes:** Above-ground biomass carbon (AGBC), Below-ground biomass carbon (BGBC), Above-ground carbon stock (AGC, AGC = AGBC) and Below-ground carbon stock (BGC, BGC = BGBC + Soil C).

## Table S2. Allometric equations for various mangrove based on DBH

| **Species** | **Equations** | **References** |
| --- | --- | --- |
| *Common equation* | 𝑊𝐴𝐺𝐵 =0.251*ρ𝐷 2.46  𝑊𝐵𝐺𝐵 =0.199*ρ0.899*𝐷 2.22 | Komiyama. *et al.* 2005  Komiyama. *et al.* 2005 |
| *Heritiera littoralis* | 𝑊𝐴𝐺𝐵 =0.251*0.84𝐷2.46  𝑊𝐵𝐺𝐵 =0.199*0.840.899*𝐷2.22 | Komiyama *et al.* 2005;  Donato *et al.* 2012 |
| *Rhizophora apiculata* | 𝑊𝐴𝐺𝐵 =0.235𝐷2.42 | Ong *et al*. 2004 |
|  | 𝑊𝐵𝐺𝐵 =0.199*0.770.899*𝐷2.22 | Komiyama *et al*. 2008 |
| *Rhizophora stylosa* | 𝑊𝐴𝐺𝐵 = 0.2206𝐷2.4292 | Clough *et al.* 1997 |
|  | 𝑊𝐵𝐺𝐵 = 0.261𝐷^1.86^ | Comley & McGuinness. 2005 |
| *Xylocarpus granatum* | 𝑊𝐴𝐺𝐵 =0.0823𝐷2.59 | Clough and Scott .1989 |
|  | 𝑊𝐵𝐺𝐵 =0.199*0.610.899*𝐷2.22 | Poungparn *et al.* 2003 |
| *Avicennia marina* | 𝑊𝐴𝐺𝐵 = 0.308𝐷^2.11^ | Comley&McGuinness. 2005 |
|  | 𝑊𝐵𝐺𝐵 = 1.28𝐷1.17 | Comley&McGuinness. 2005 |
| *Bruguiera gymnorrhiza* | 𝑊𝐴𝐺𝐵 = 0.186𝐷^2.31^ | Clough & Scott. 1989 |
|  | 𝑊𝐵𝐺𝐵 = 0.4697𝐷1.5543 | Liao *et al*. 1991 |
| *Sonneratia apetala* | 𝑊𝐴𝐺𝐵 = 0.28(𝐷^2^𝐻)^0.693^ | Liu *et al*.2014 |
|  | 𝑊𝐴𝐺𝐵 = 0.038(𝐷^2^𝐻)^0.759^ | Liu *et al*.2014 |
| *Lianas plants* | 𝑊𝐵=𝐷2.657*e0.968*ln𝐷 | Schnitzer *et al.* 2006 |
| *Excoecaria agallocha* | 𝑊𝐴𝐺𝐵 =0.251*0.41𝐷2.46  𝑊𝐵𝐺𝐵 =0.199*0.410.899*𝐷2.22 | Komiyama *et al.* 2005;  Donato *et al.* 2012 |
| *Kandelia obovata* | 𝑊𝐴𝐺𝐵 = 651.63(𝐷^2^𝐻)^1.053^ | Tam *et al.* 1995 |
|  | 𝑊𝐵𝐺𝐵 = 271.02(𝐷^2^𝐻)^0.990^ | Tam *et al*. 1995 |
| *Ceriops tagal* | 𝑊𝐴𝐺𝐵 =0.251*0.746*𝐷2.46 | Komiyama. *et al.* 2005 |
|  | 𝑊𝐵𝐺𝐵 =0.199*0.7460.899*𝐷2.22 | Komiyama. *et al.* 2005 |
| *Aegiceras corniculatum* | 𝑊𝐴𝐺𝐵 = 31.34(𝐷^2^𝐻)^0.465^ | Tam *et al*. 1995 |
|  | 𝑊𝐵𝐺𝐵 = 9.33(𝐷^2^𝐻)^0.303^ | Tam *et al*. 1995 |
| *Laguncularia racemosa* | 𝑊𝐴𝐺𝐵 = 0.362𝐷^1.93^  𝑊𝐵𝐺𝐵 =0.199*0.60.899*𝐷2.22 | Kauﬀman&Donato. 2012  Kauﬀman&Donato. 2012 |
| *Aegiceras corniculatum* | 𝑊𝐴𝐺𝐵 = 31.34(𝐷^2^𝐻)^0.465^ | Tam *et al*. 1995 |
|  | 𝑊𝐵𝐺𝐵 = 9.33(𝐷^2^𝐻)^0.303^ | Tam *et al*. 1995 |

**Notes:** W means tree weight, ρ means wood density, 𝐷 means diameter breast height (DBH) and 𝐻 means tree height.

**Table S3.** The main results of AGC and BGC relation fitting by using the optimization model

|  | **Linear** | **Polynomial** | **Logarithm** | **Exponential** |
| --- | --- | --- | --- | --- |
| **Formula** | BGC=a+b*AGC | BGC=a+b*AGC+c*AGC^2^ | BGC=a+b*ln(AGC+c) | BGC=a*exp^b*AGC^ |
| **Latitude Regions** |  |  |  |  |
| FJP | R^2^=0.70, P<0.01 | R^2^=0.70, P<0.01 | Not convergent | Not convergent |
| GDP | R^2^=0.60, P<0.01 | R^2^=0.62, P<0.01 | Not convergent | R^2^=0.64, P<0.01 |
| HNN | R^2^=0.60, P<0.01 | R^2^=0.59, P<0.01 | R^2^=0.59, P<0.01 | R^2^=0.52, P<0.01 |
| HNS | R^2^=0.50, P<0.01 | R^2^=0.45, P<0.05 | R^2^=0.45, P<0.01 | Not convergent |
| **Tidal Types** |  |  |  |  |
| DT | R^2^=0.41, P<0.01 | R^2^=0.52, P<0.01 | R^2^=0.36, P<0.01 | R^2^=0.46, P<0.01 |
| ST | R^2^=0.70, P<0.01 | R^2^=0.70, P<0.01 | R^2^=0.64, P<0.01 | Not convergent |
| IDT | R^2^=0.58, P<0.01 | R^2^=0.58, P<0.01 | R^2^=0.58, P<0.01 | Not convergent |
| IST | R^2^=0.22, P<0.01 | R^2^=0.18, P<0.01 | R^2^=0.17, P<0.01 | Not convergent |
| **Domain Species** |  |  |  |  |
| Bs | R^2^=0.74, P<0.01 | R^2^=0.73, P<0.01 | R^2^=0.72, P<0.01 | Not convergent |
| Rs | R^2^=0.30, P<0.01 | R^2^=0.33, P<0.01 | R^2^=0.22, P<0.01 | Not convergent |
| Ac | R^2^=0.24, P<0.01 | R^2^=0.18, P<0.01 | R^2^=0.14, P<0.01 | Not convergent |
| Am | R^2^=0.28, P<0.01 | R^2^=0.16, P<0.01 | R^2^=0.15, P<0.01 | R^2^=0.21, P<0.01 |

**Notes:** The optimal model was used to fit AGC and BGC in different ways, and the correlation with the strongest fitting degree and best applicability was selected. R^2^ is the correlation coefficient and P is the statistical probability value.

**Table S1. Reference:**

Adame, M. F., J. B. Kauffman, I. Medina, J. N. Gamboa, O. Torres, J. P. Caamal, M. Reza, and J. A. Herrera-Silveira. 2013. Carbon stocks of tropical coastal wetlands within the karstic landscape of the Mexican Caribbean. Plos One **8**.

Alongi, D., F. Tirendi, and B. Clough. 2000. Below-ground decomposition of organic matter in forests of the mangroves Rhizophora stylosa and Avicennia marina along the arid coast of Western Australia. Aquatic Botany **68**:97-122.

Donato, D. C., J. B. Kauffman, D. Murdiyarso, S. Kurnianto, M. Stidham, and M. Kanninen. 2011. Mangroves among the most carbon-rich forests in the tropics. Nature Geoscience **4**:293-297.

Dung, L. V., N. T. Tue, M. T. Nhuan, and K. Omori. 2016. Carbon storage in a restored mangrove forest in Can Gio Mangrove Forest Park, Mekong Delta, Vietnam. Forest Ecology and Management **380**:31-40.

He, Q., W. Zheng, X. Huang, X. Liu, W. Shen, and F. He. 2017. Carbon storage and distribution of mangroves at Qinzhou bay. Journal of Central South University of Forestry and Technology **37**:121-126.

Liu, H., H. Ren, D. Hui, W. Wang, B. Liao, and Q. Cao. 2014. Carbon stocks and potential carbon storage in the mangrove forests of China. Journal of Environmental Management **133**:86-93.

Lunstrum, A., and L. Chen. 2014. Soil carbon stocks and accumulation in young mangrove forests. Soil Biology and Biochemistry **75**:223-232.

Murdiyarso, D., J. Purbopuspito, J. B. Kauffman, M. W. Warren, S. D. Sasmito, D. C. Donato, S. Manuri, H. Krisnawati, S. Taberima, and S. Kurnianto. 2015. The potential of Indonesian mangrove forests for global climate change mitigation. Nature Climate Change **5**:1089-1092.

Nam, V. N., S. D. Sasmito, D. Murdiyarso, J. Purbopuspito, and R. A. MacKenzie. 2016. Carbon stocks in artificially and naturally regenerated mangrove ecosystems in the Mekong Delta. Wetlands ecology and management **24**:231-244.

Peng, C., J. Qian, X. Guo, H. Zhao, N. Hu, Q. Yang, C. Chen, and L. Chen. 2016. Vegetation carbon stocks and net primary productivity of the mangrove forests in Shenzhen, China. Ying yong sheng tai xue bao= The journal of applied ecology **27**:2059-2065.

Wang, G., D. Guan, M. R. Peart, Y. Chen, and Y. Peng. 2013. Ecosystem carbon stocks of mangrove forest in Yingluo Bay, Guangdong Province of South China. Forest Ecology and Management **310**:539-546.

**Table S2. Reference:**

Clough, B., Dixon, P. & Dalhaus, O. (1997). Allometric relationships for estimating biomass in multi- stemmed mangrove trees. Australian Journal of Botany, 45, 1023-1031.

Clough, B. & Scott, K. (1989). Allometric relationships for estimating above-ground biomass in six mangrove species. Forest ecology management, 27, 117-127.

Comley, B. & McGuinness, K. (2005). Above-and below-ground biomass, and allometry, of four common northern Australian mangroves. Australian Journal of Botany, 53, 431-436.

Kauffman, J.B. & Donato, D.C. (2012). Protocols for the measurement, monitoring and reporting of structure, biomass, and carbon stocks in mangrove forests. Citeseer.

Komiyama, A., Ong, J.E. & Poungparn, S. (2008). Allometry, biomass, and productivity of mangrove forests: A review. Aquatic Botany, 89, 128-137.

Komiyama, A., Poungparn, S. & Kato, S. (2005). Common allometric equations for estimating the tree weight of mangroves. Journal of Tropical Ecology, 21, 471-477.

Liu, H., Ren, H., Hui, D., Wang, W., Liao, B. & Cao, Q. (2014). Carbon stocks and potential carbon storage in the mangrove forests of China. Journal of Environmental Management, 133, 86-93.

Ong, J., Gong, W. & Wong, C. (2004). Allometry and partitioning of the mangrove, Rhizophora apiculata.

Forest Ecology Management, 188, 395-408.

Pendleton, L., Donato, D.C., Murray, B.C., Crooks, S., Jenkins, W.A., Sifleet, S. et al. (2012). Estimating global “blue carbon” emissions from conversion and degradation of vegetated coastal ecosystems. 7.

Poungparn, S., Komiyama, A., Patanaponpaipoon, P., Jintana, V., Sangtiean, T., Tanapermpool, P. et al. (2003). Site-independent allometric relationships for estimating above-ground weights ofmangroves. Journal of Tropical Ecology, 12, 147-158.

Schnitzer, S.A., DeWalt, S.J. & Chave, J. (2006). Censusing and Measuring Lianas: A Quantitative Comparison of the Common Methods. Biotropica, 38, 581-591.

Songfa, Baowen, L., De, Z. & Zheng., Z. (1991). Biomass and Productivity of Bruguiera gymnorrhiza Stand. Forest Research, 1.

Tam, N., Wong, Y., Lan, C. & Chen, G. (1995). Community structure and standing crop biomass of a mangrove forest in Futian Nature Reserve, Shenzhen, China. Hydrobiologia, 295, 193-201.
